# Supplementary material for: The co-chaperone Fkbp5 shapes the acute stress response in the paraventricular nucleus of the hypothalamus of male mice
Source: Mol Psychiatry. 2021 Mar 1;26(7):3060–76. doi: 10.1038/s41380-021-01044-x (PMC8505251; doi:10.1038/s41380-021-01044-x)
Supplement: Supplementary file 1 — Supplemental Material [file 41380_2021_1044_MOESM1_ESM.docx]

**Supplementary figures.**


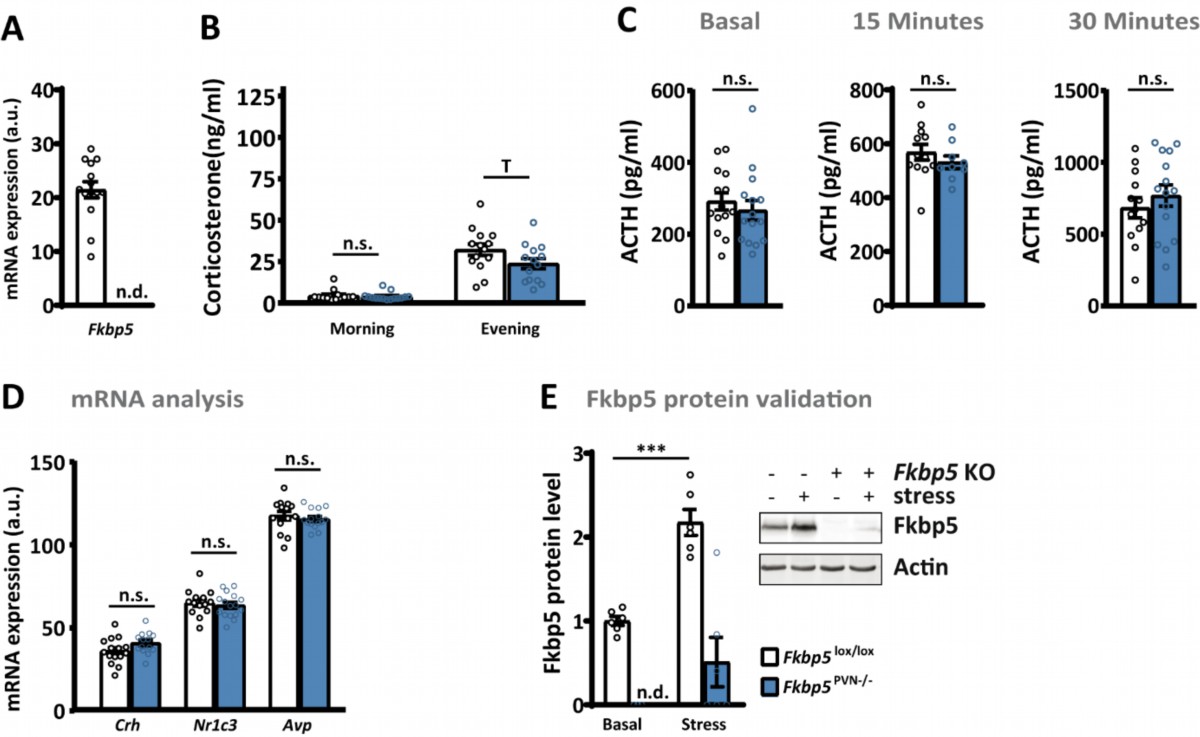


**Supplementary Figure 1: Corticosterone and ACTH levels of *Fkbp5*^PVN-/-^ mice (16-20 weeks of age). (A)** Validation of the *Fkbp5* deletion in *Fkbp5*^PVN-/-^ *animals* (*Fkbp5*^PVN-/-^ n = 16; *Fkbp5*^lox/lox^ n =15)*.* **(B)** *Fkbp5* deletion in the PVN has no effect on basal CORT levels (*Fkbp5*^PVN-/-^ n = 16; *Fkbp5*^lox/lox^ n =15). **(C)** ACTH levels under basal,15 and 30 minutes after stress onset were unaltered (*Fkbp5*^PVN-/-^ n = 9-16; *Fkbp5*^lox/lox^ n =12-15). **(D)** mRNA changes of stress responsive genes within the PVN under basal conditions (*Fkbp5*^PVN-/-^ n = 16; *Fkbp5*^lox/lox^ n =15). **(E)** Fkbp5 protein levels under basal and stress conditions (*Fkbp5*^PVN-/-^ n = 6; *Fkbp5*^lox/lox^ n = 6 for stressed and non-stressed group). All data were received from mice between 16 and 20 weeks of age and are presented as mean ± SEM and were analyzed with a student’s t-test (A-D) or with a two way ANOVA (E). n.d. = not detectable; n.s. = not significant; T = 0.05 < p < 0.1; *** = p < 0.001.


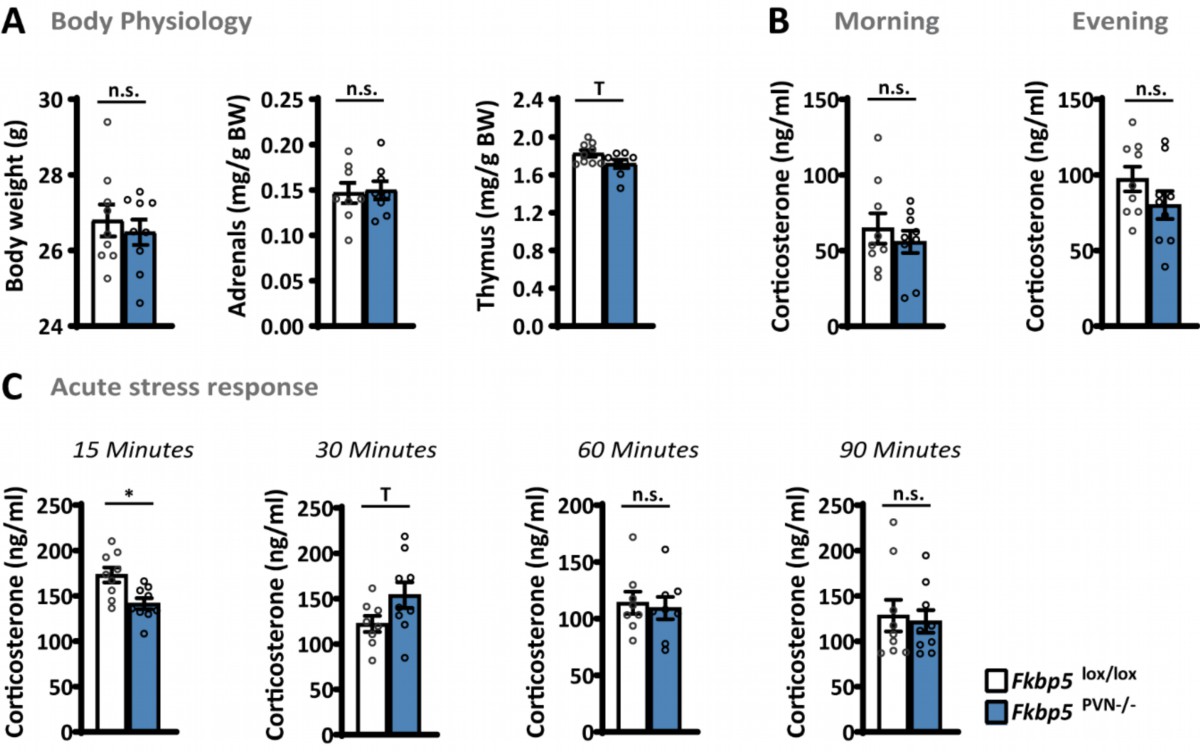


**Supplementary Figure 2: Young mice with *Fkbp5* deletion in the PVN**. **(A)** Animals with an age of 8-10 weeks had no alterations in body physiology. **(B)** Morning and evening corticosterone levels were unchanged. **(C)** FKBP51^PVN-/-^ animals had significantly lower corticosterone levels 15 minutes after stress onset compared to the control group (group size for A-C: *Fkbp5*^PVN-/-^ n = 9; *Fkbp5*^lox/lox^ n =9). All data were received from mice between 8 and 10 weeks of age and are presented as mean ± SEM. Data were analyzed with a student’s t-test. n.s. = not significant; T = 0.05 < p < 0.1; * = p < 0.05.


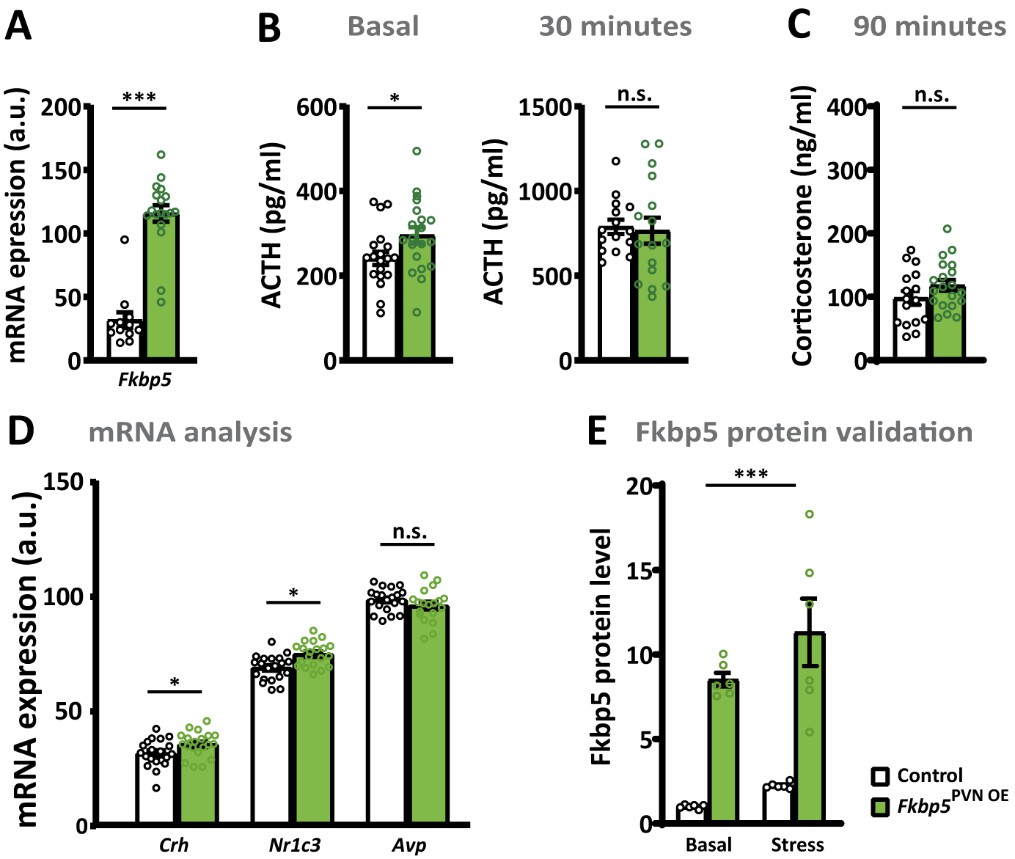


**Supplementary Figure 3: Overexpression of *Fkbp5* in the PVN affected ACTH and mRNA levels. (A)** *Fkbp5* overexpression resulted in a significant increase of *Fkbp5* mRNA (n (control) = 12 vs n (*Fkbp5*^PVN OE^) = 18). **(B)** ACTH levels were significantly higher in *Fkbp5*^PVN OE^ mice under basal conditions (n = 20 vs. 20) and unchanged 30 minutes after stress onset (n = 12 vs. 12) compared to their controls**. (C)** We did not detect any differences in corticosterone levels 60 minutes after stress onset (n = 20 vs. 20). **(D)** mRNA levels of *N1c3* and *Crh* and *Avp* under basal conditions (n = 20 vs. 20). **(E)** Viral overexpression resulted in a 4-fold Fkbp5 protein upregulation (n = 6 vs. 6 for stress and non-stressed groups). All data were received from mice between 14-20 weeks of age and are presented as mean ± SEM and were analyzed with a student’s t-test (A-D) or with a two way ANOVA (E). n.s. = not significant; * = p < 0.05, *** = p < 0.001.


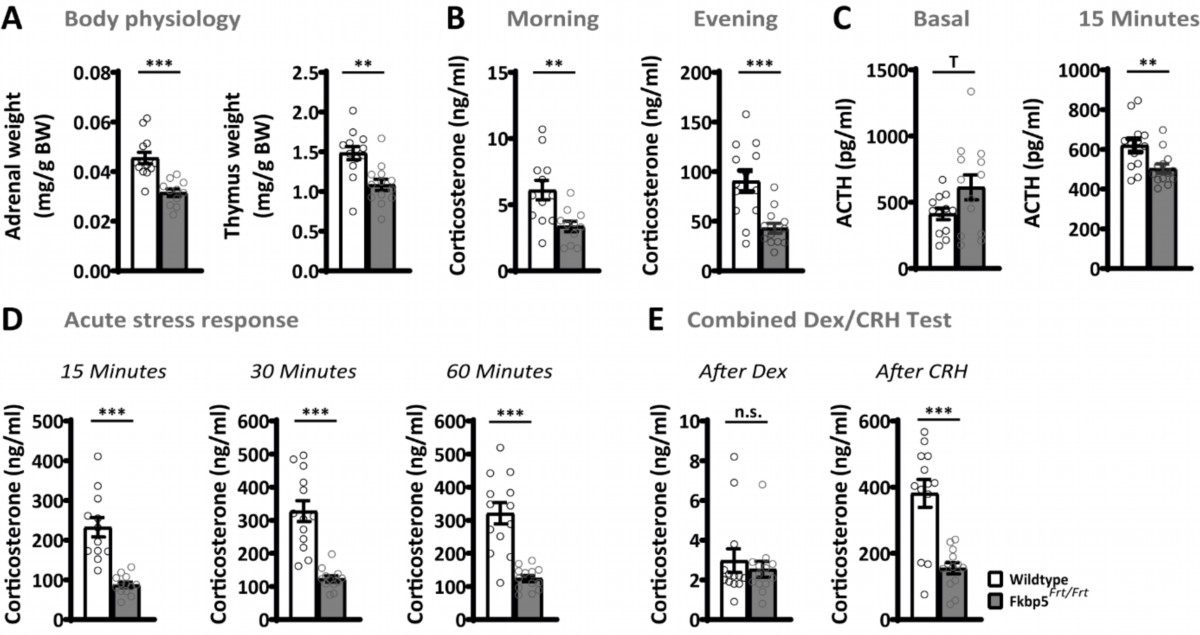


**Supplementary Figure 4: Validation of the *Fkbp5*^Frt/Frt^ mouse line in comparison to wildtype littermates. (A)** Adrenal and thymus weights on sacrifice day. **(B)** Morning and evening corticosterone levels. **(C)** ACTH under basal conditions and 15 minutes after stress onset**. (D)** Corticosterone after 15 minutes of restrain stress. **(E)** Combined Dex/CRH test. For all groups, n (wildtype) = 12 vs. n (*Fkbp5*^Frt/Frt^) = 11). All data were received from mice of 16-24 weeks of age and are presented as mean ± SEM and were analyzed with a student’s t-test. n.s. = not significant; T = 0.05 < p < 0.1; ** = p < 0.01, *** = p < 0.001.


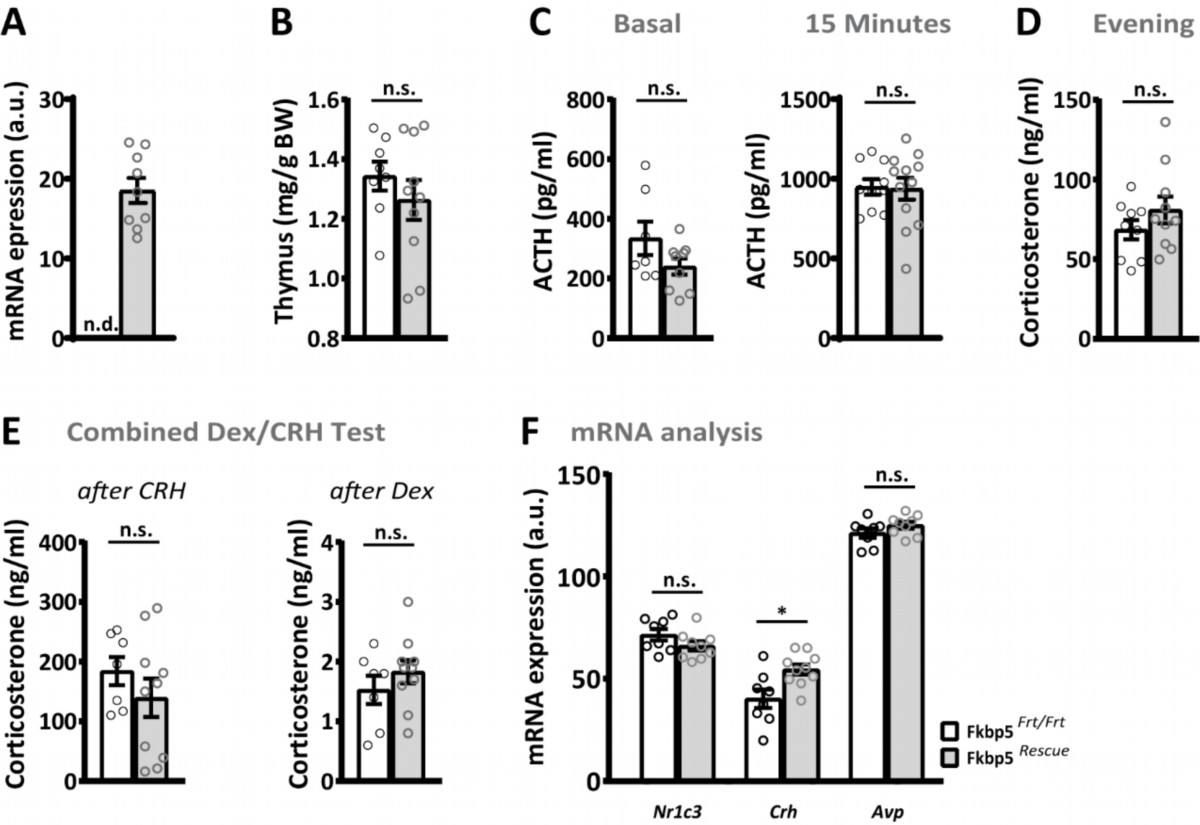


**Supplementary Figure 5: Corticosterone and ACTH levels of *Fkbp5*^Rescue^ mice. (A)** Validation of *Fkbp5* mRNA expression compared to *Fkbp5*^Frt/Frt^. **(B)** *Fkbp5* reinstatement had no effect on thymus weights. **(C)** ACTH hormone levels were unaltered under basal and 15 minutes after stress. **(D)** Evening corticosterone. **(E)** Rescue of endogenous *Fkbp5* in global knock-out animals had no significant effect on the Dex/CRH test. **(F)** mRNA levels of stress responsive genes under basal conditions. Group sizes for A-F: *Fkbp5*^Rescue^ n = 10; *Fkbp5*^Frt/Frt^ n =9). All data were received from mice between 16 and 20 weeks of age and are presented as mean ± SEM and were analyzed with a student’s t-test. n.s. = not significant; n.d. = not detectable; * = p < 0.05.


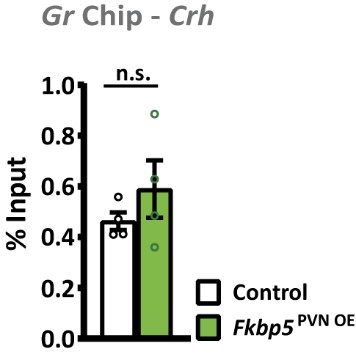


**Supplementary Figure 6: GR to GRE binding within the *Crh* gene after stress.** Mice were sacrificed 30 minutes after stress onset. Every n consists of a pool of 4 individual hypothalami. All data were received from mice aged between 12-16 weeks and are indicated as mean ± SEM and were analyzed with a student’s t-test; (n = 4 vs. 4); n.s. = not significant.


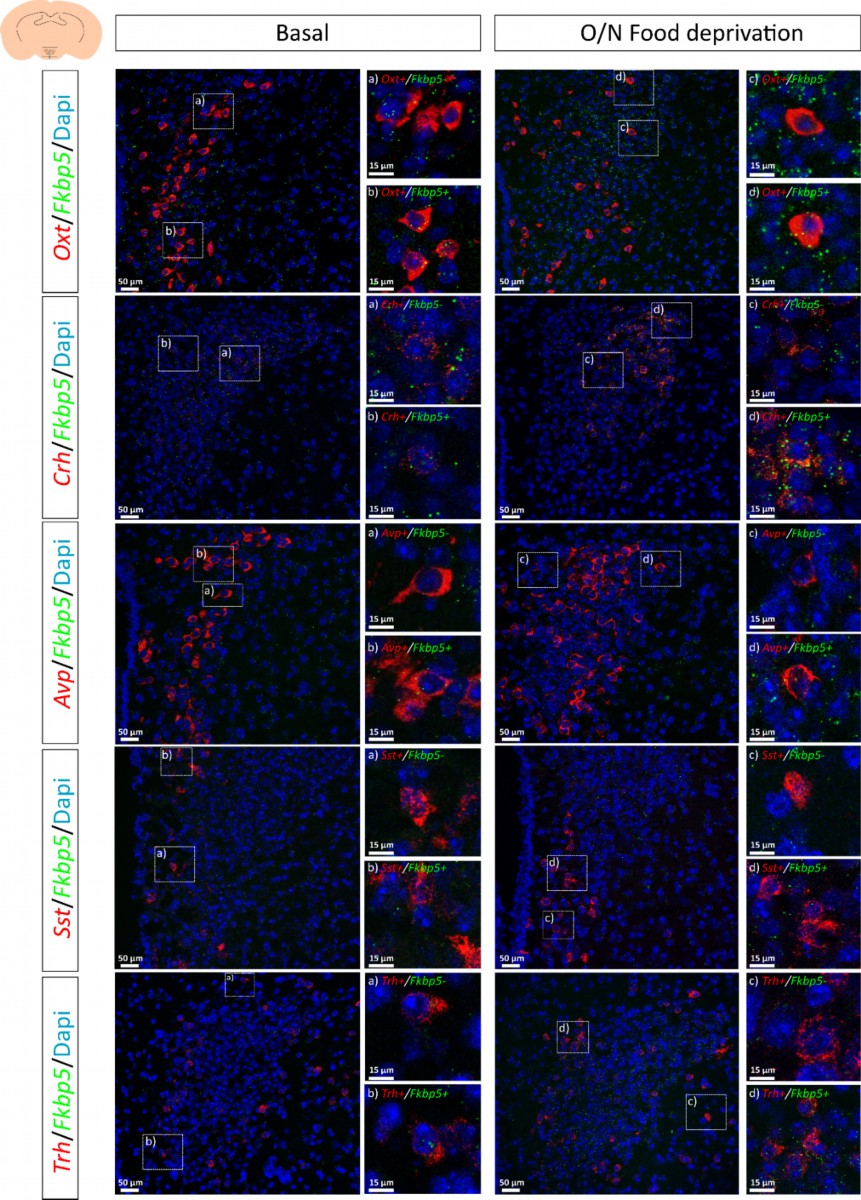


**Supplementary Figure 7: RNAscope analysis of *Fkbp5* expression in stress response neuronal cell populations of C57Bl/6** **under basal and stressed conditions.** In comparison to single-cell sequencing, RNAscope revealed a significant higher co-localization ratio of *Fkbp5* in oxytocin (*Oxt*), corticotropin-releasing hormone (*Crh*), vasopressin (*Avp*), somatostatin (*Sst*) and thyronine releasing hormone (*Trh*) neurons. Overnight food deprivation increased *Fkbp5* mRNA in all cell populations.


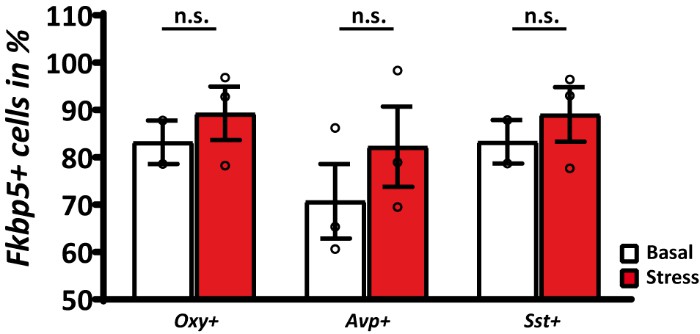


**Supplementary Figure 8: Quantification of *Fkbp5* co-localization in stress response markers under basal and stress conditions.** Under basal conditions *Fkbp5* mRNA signal was detected in 83% of *Oxt*+ neurons, 68% in *Avp*+ and 84% of *Sst*+ neurons. We monitored a non-significant increase in *Fkbp5* mRNA expression in all neuronal populations. Each n represents an average of the detected *Fkbp5*+ cells within six z-stacks of 1um each (3 per PVN side). Data are were received from animals between 8-12 weeks of age and are presented as mean ± SEM and were analyzed with a student’s t-test. n.s. = not significant.


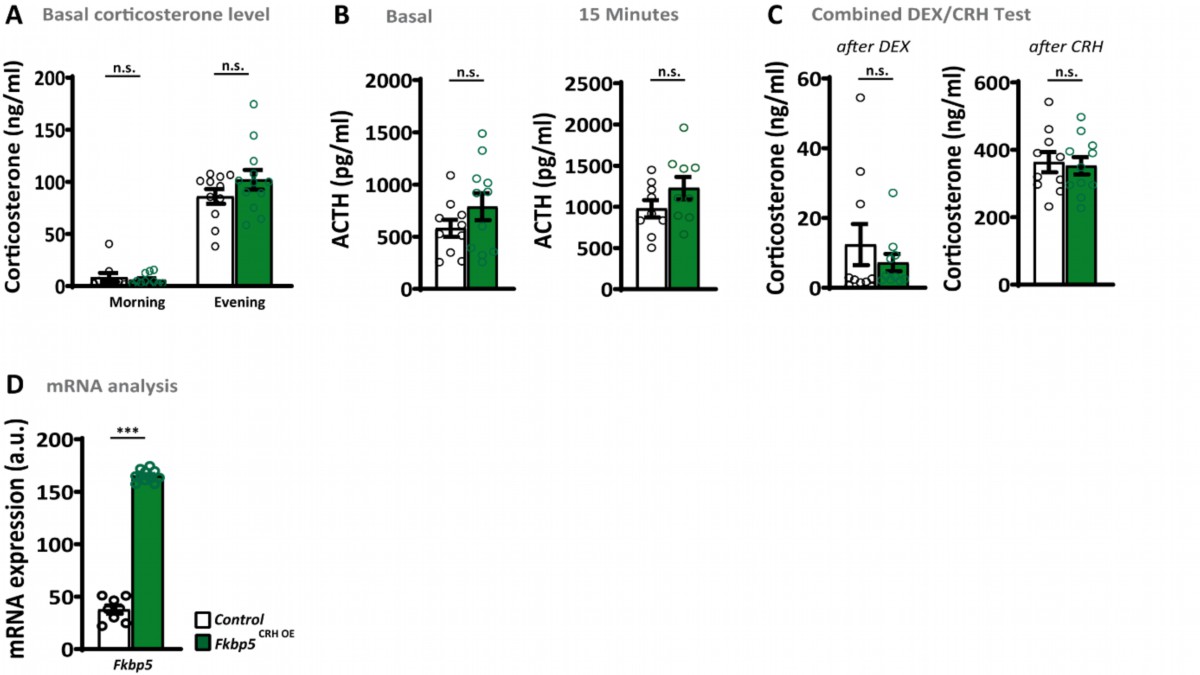


**Supplementary Figure 9: Corticosterone and ACTH levels of *Fkbp5*^CRH OE^ mice. (A)** *Fkbp5* overexpression in CRH neurons within the PVN had no effect on basal morning (*Fkbp5*^CRH OE^ n = 11; *Control* n = 9) and evening (*Fkbp5*^CRH OE^ n = 12; Control n = 11) CORT level. **(B)** ACTH level were unaltered at baseline (*Fkbp5*^CRH OE^ n = 11; Control n = 10) and 15 minutes post stress (*Fkbp5*^CRH OE^ n = 9; Control n = 9). **(C)** In the combined Dex/CRH test CORT level were unaltered in *Fkbp5*^CRH OE^ (n_after DEX_ = 10; n_after CRH_ = 11) compared to controls (n = 10) **(D)** *Fkbp5* mRNA expression level. All data are presented as mean ± SEM and were analyzed with a student’s t-test. n.s. = not significant; *** = p < 0.001.
